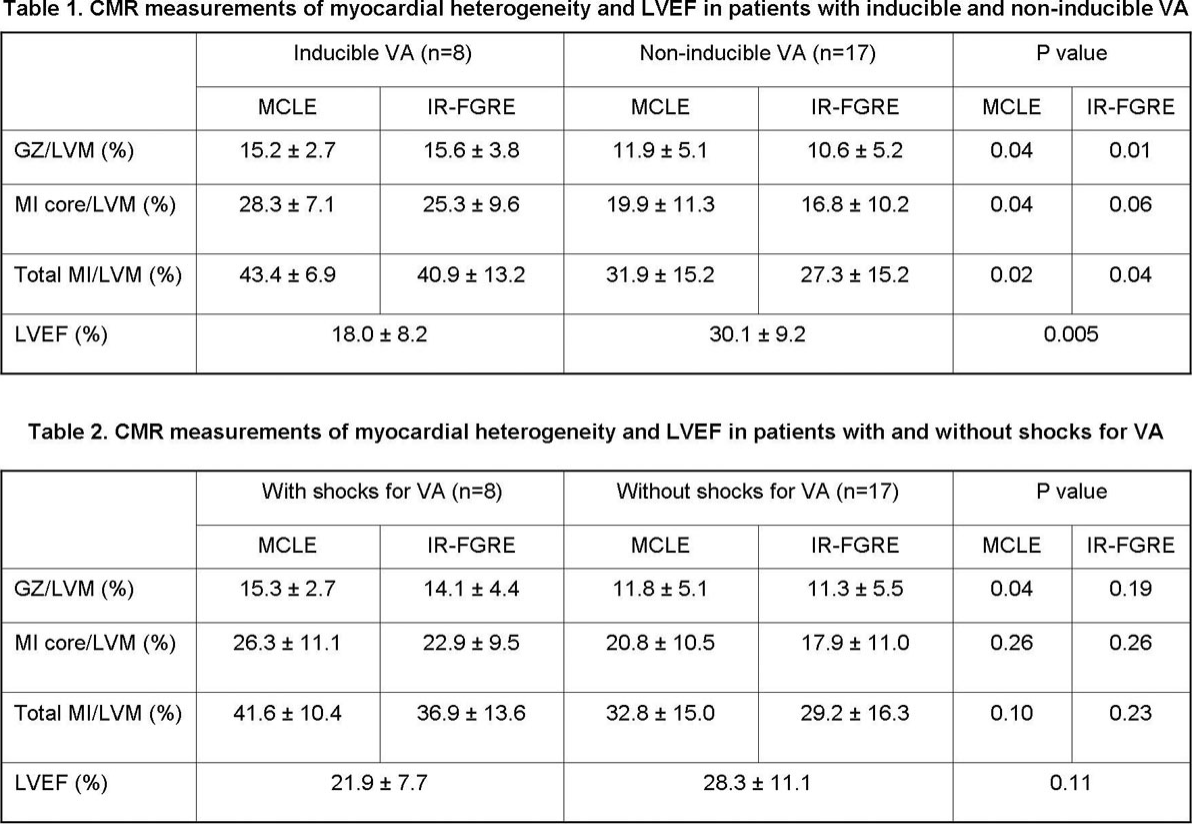# CMR measurements of myocardial infarct heterogeneity using MCLE and IR-FGRE: correlation with arrhythmia inducibility and severe ICD events in patients with ischemic heart disease

**DOI:** 10.1186/1532-429X-14-S1-O105

**Published:** 2012-02-01

**Authors:** Yuesong Yang, Kim A Connelly, Tawfiq Zeidan, Subodh B Joshi, John J Graham, Gideon A Paul, Rhonda Walcarius, Alexander J Dick, Eugene Crystal, Graham Wright

**Affiliations:** 1 Imaging Research and Cardiology, Sunnybrook Health Sciences Centre, Toronto, ON, Canada; 2 Cardiology, St. Michael Hospital, Toronto, ON, Canada; 3 Cardiology, Ottawa Heart Institute, Ottawa, ON, Canada

## Summary

This study used CMR to evaluate patients with IHD prior to ICD implantation and correlated CMR measurements to VA inducibility and spontaneous VA events during follow-up. The results demonstrated that the gray-zone measurement using MCLE may be more sensitive in predicting appropriate ICD therapy for VA.

## Background

In addition to measures of left ventricular ejection fraction (LVEF) and clinical staging of heart failure, myocardial infarct (MI) heterogeneity including MI and peri-infarct gray-zone (GZ) has the potential to predict the occurrence of inducible sustained ventricular arrhythmia (VA) and spontaneous VA events after implantation of implantable defibrillator (ICD) in patients with ischemic heart disease (IHD). Late-gadolinium (Gd)-enhancement (LGE) cardiac MR (CMR) using inversion-recovery fast-gradient-echo (IR-FGRE) is commonly used for the determination of infarct heterogeneity in these patients. Recently, a multi-contrast late-enhancement (MCLE) sequence has been developed for better infarct heterogeneity quantification. Compared to IR-FGRE, we hypothesized that MCLE may be a more sensitive method to predict the occurrence of inducible VA and severe events post-ICD implantation.

## Methods

This study used CMR to evaluate patients with IHD prior to ICD implantation and correlated CMR measurements to VA inducibility and spontaneous VA events during follow-up. The MRI protocol included LV functional parameter assessment using steady-state free precession (SSFP), as well as LGE-MRI using IR-FGRE and MCLE post double-dose Gd injection. LV functional parameters were measured using Q-Mass or CMR42 software. The GZ analysis in IR-FGRE used a full-width half-maximum method. For MCLE, GZ analysis used a semi-automated data clustering algorithm. An unpaired t-test with unequal variance was used for the statistical analysis of the proportion of GZ, MI core and total MI relative to LV myocardium mass.

## Results

Twenty-five patients with IHD for planned ICD implantation (age 64 +/- 10 yrs, 88% men, average LVEF 26.2 +/- 10.4%, 40% secondary prevention) were enrolled. All patients completed the MRI protocol, ventricular electro-physiology study (EPS), and six months follow-up at the ICD clinic. Eight patients (32%) had inducible VA during programmed ventricular stimulation and six of these eight patients had at least one appropriate shock for VF from the ICD at follow-up. Another two patients without inducible VA had appropriate shocks from the ICD. CMR measurements of LVEF and myocardial infarct heterogeneity are shown in Tables 1&1(2). LVEF and GZ measurements from IR-FGRE and MCLE showed a statistically significant difference between groups of inducible and non-inducible VA; however, only the proportion of GZ measured with MCLE demonstrated a statistical difference (P=0.04) between patients with and without ICD shocks for VA.

## Conclusions

CMR measurements of myocardial heterogeneity using MCLE and IR-FGRE are good predictors for the occurrence of inducible VA; however, the gray-zone measurement using MCLE may be more sensitive in predicting appropriate ICD therapy for VA.

## Funding

CIHR.

**Figure 1:**